# Miltirone promotes pyroptosis via increasing pyroptosis-related protein NLRP3 and AIM2 in kidney renal clear cell carcinoma

**DOI:** 10.3389/fimmu.2026.1702901

**Published:** 2026-02-23

**Authors:** Tao Huang, Qinghai Wang, Yang Gao, Hongyang Wang, Chen Guo, Lixia Song, Pingli He, Jinzhen Cai

**Affiliations:** 1 Department of Kidney Transplantation, The Affiliated Hospital of Qingdao University, Qingdao, Shandong, China; 2 Department of Anesthesiology, The Affiliated Hospital of Qingdao University, Qingdao, Shandong, China; 3 Department of Organ Transplant Center, The Affiliated Hospital of Qingdao University, Qingdao, Shandong, China

**Keywords:** immune, kidney renal clear cell carcinoma, miltirone, prognosis, pyroptosis

## Abstract

**Background:**

Pyroptosis, a type of programmed cell death, exerts direct influence on inflammatory processes and immune response. A previous study suggests that miltirone exhibits notable anti-tumor activities and has been shown to induce tumor cell pyroptosis. Nevertheless, the therapeutic value of miltirone in kidney renal clear cell carcinoma (KIRC) remains underexplored.

**Methods and results:**

Using TCGA pan-cancer data, we uncovered widely expressed pyroptosis-related genes (NLRP3 and AIM2). Mechanistically, the genomic amplification alteration and high CNV percentages in pan-cancer induced an abnormal expression. StromalScore analysis suggested that NLRP3 and AIM2 were activated by the tumor immune microenvironment in KIRC. Enrichment analysis indicated that NLRP3 and AIM2 regulated the inflammatory response and were related to immune infiltration in KIRC. Furthermore, Kaplan–Meier curve and ROC analysis indicated that a high expression of AIM2 was associated with a worse prognosis of KIRC patients. MTT assays and flow cytometry revealed that miltirone treatment induced KIRC cell pyroptosis and inhibited cell proliferation, with changes in the expression level of NLPR3, AIM2, caspase-3, and GSDMD. The enhancement of cell pyroptosis and the release of IL-1β and IL-18 cytokines were reversed by pretreatment with the pyroptosis inhibitor VX-765.

**Conclusions:**

Our study revealed the prognostic value of NLRP3 and AIM2 in KIRC. Miltirone treatment inhibited KIRC cell proliferation and enhanced cell pyroptosis via the NLRP3/AIM2/GSDMD axis. This study provides a novel molecular mechanism and potential therapeutic targets in KIRC progression.

## Introduction

Kidney renal clear cell carcinoma (KIRC) accounts for more than 75%–85% of primary renal cell adenocarcinomas and is one of the major cancers of the genitourinary system (1). A total of 60% of KIRC patients are diagnosed with tumor metastasis at the initial diagnostic phase, and about a quarter of patients experience relapse and metastasis after radical nephrectomy (2). The average survival of KIRC patients after metastasis is 1.5 years, and the survival rate to 5 years is less than 10% (3). Therefore, a comprehensive understanding of the pathogenesis and biomarkers of KIRC will help to explore novel drugs or molecular targets.

Miltirone (C_19_H_22_O_2_, molecular weight: 282.38), a natural compound with significant biological activity, has garnered attention in recent years for its potential therapeutic applications (4). Derived from various plant sources, miltirone is known for its antioxidant properties and its ability to modulate cellular pathways (4–6). Increasing evidence indicated that miltirone induces cancer cell apoptosis and pyroptosis, two critical forms of programmed cell death (6, 7). *In vitro* studies have shown that miltirone inhibits cell growth and promotes pyroptosis in hepatocellular carcinoma (7). However, how miltirone affects KIRC cells and its exact mechanism remain to be fully explored.

Unlike other cell death models, pyroptosis is a type of programmed cell lytic death characterized by inflammasome-induced caspase-1 activation and GSDM protein cleavage, resulting in cell membrane perforation, cell swelling, and release of inflammatory cytokines (IL-1β, IL-18) (8–10). Several studies have indicated that pyroptosis is critical for the occurrence and development of tumors, with potential applications in tumor treatment strategies (11, 12). The family of NOD-like receptor (NLR) proteins, recognized as pattern recognition receptors (PRRs), has emerged as a key factor in triggering the primary innate immune response to stress and cell damage (13). The 14 NLR proteins are distinguished by their N-terminal pyrin domain and identified for their ability to assemble into larger inflammasome structures (14). Studies of NLRs have shown the importance of NLRP3 upregulation and its potential as a therapeutic target in KIRC (15). Other studies that have emerged offer contradictory findings about the low expression level and anti-cancer function of NLRP3 in KIRC (16). To date, however, much uncertainty still exists about the immunological function and prognostic significance of NLRs in KIRC.

This study provides an in-depth investigation into the functional effects of miltirone on the progression of KIRC, with a focus on uncovering the molecular pathways involved in KIRC pyroptosis. In this research, we profiled the public TCGA data to evaluate the expression levels and genetic alterations of pyroptosis-related genes across 22 pan-cancer studies, with a particular focus on the immunological function and prognostic value of NLRP3 and absent in melanoma 2 (AIM2) in KIRC. Moreover, we conducted *in vitro* experiments to validate our findings and further understand the mechanistic role of miltirone treatment in KIRC cell pyroptosis and molecular mechanism.

## Materials and methods

### Investigating the expression levels of NLRs and AIM2 among pan-cancers

The Gene Set Cancer Analysis (GSCALite) platform, compiles an extensive collection exceeding 11,000 tumor samples from the TCGA database (https://portal.gdc.cancer.gov). This study utilized the GSCALite platform to examine how NLRs and AIM2 mRNA expressions differ between tumor tissues and normal tissues through the “tumor vs. normal” analysis module. Additionally, RNA sequencing analysis was conducted on data sourced from both the Genotype-Tissue Expression (GTEx) project and TCGA RNAseq database. This analysis was performed in the FPKM format, utilizing R software (version 4.1.0) alongside the limma package for statistical analysis. Furthermore, data on NLRs and AIM2 protein expression across pan-cancer were gathered from the Human Protein Atlas (HPA) database, accessible at https://www.proteinatlas.org, employing immunohistochemistry (IHC) analysis.

### Genetic alterations of NLR family and AIM2 among pan-cancer

We next assessed whether the differential expression of NLR family and AIM2 was preserved in genetic structure, focusing on genetic alteration frequency and copy number variations (CNV). The cBioPortal for Cancer Genomics, available at https://www.cbioportal.org/, was used to perform mutation and CNV analysis. Firstly, we browsed available datasets and selected the “pan-cancer analysis of whole genomes (ICGA/TCTA)” study, which included 2,922 samples for visualization and analysis. Next, we selected the genomic profiles for “mutations” and “consensus putative gene level copy number calls”. Then, 2,683 samples with mutation and CNA data were selected for gene query. The “cancer types summary” module showed the alteration frequency of each gene among pan-cancers.

### Correlation analysis of NLRs/AIM2 levels with tumor microenvironment in pan-cancer

TCGA-RNA seq data were download from the TCGA database and converted into TPM format. Using log2(value+1), we removed normal and clinically uninformative samples. Next, R (version 4.1.0) and estimate package were used to analyze the correlation between NLRs/AIM2 levels and immune score in pan-cancer.

### PPI network construction and enrichment analysis

TCGA-KIRC RNA seq data were downloaded from the TCGA database and subjected to R software (version 3.3.6) for correlation analysis. Spearman correlation was applied in the analysis of NLRs/AIM2 expression in KIRC, and *p* = 0.05 was considered significant. The data were visualized using the “ggplot2” package with heat map. In addition, we searched STRING by multiple protein names at the homepage. The network view predicted associations between the specified proteins. The DAVID functional annotation tool was used to elucidate the biological significance of the NLR family and AIM2 gene.

### Analysis of immune infiltration in KIRC

For the assessment of KIRC specimens, the CIBERSORT algorithm was employed to identify eight distinct subtypes of immune cells. The correlation between NLRs/AIM2 gene expression and the levels of immune infiltration was investigated using the TIMER database, which provided immune infiltration scores. We carried out Spearman’s correlation analysis in order to assess the association between genes and immune infiltration.

### Tumor–immune interaction analysis

Tumor–immune interaction analysis was performed at the TISIDB website (http://cis.hku.hk/TISIDB/index.php) to evaluate NLRs/AIM2 gene expression across immune subtypes. The gene symbols were searched at the TISIDB. The “subtype” module showed the correlation between NLRs/AIM2 expression levels and six immune subtypes across KIRC, including wound healing (C1), IFN-gamma dominant (C2), inflammatory (C3), lymphocyte depleted (C4), immunologically quiet (C5), and TGF-beta dominant (C6).

### Survival analysis

Kaplan–Meier plots were utilized to perform overall survival analysis in KIRC. The clinical data were stratified into two groups, namely, high-expression and low-expression groups, based on the median value of the gene expression levels. To show the relationship between sensitivity and specificity and evaluate diagnostic tests, ROC analysis was carried out by utilizing the pROC package, and the results were visualized by utilizing ggplot2. The survival package was used to test the proportional risk hypothesis and to perform Cox regression analysis. Nomogram-related models were constructed and visualized using the RMS package.

### Cell culture and treatment

Four kinds of KIRC cells (786-O, ACHN, A498, and Caki-2) were obtained from ATCC (CA, USA) and utilized for the cell experiment research. The KIRC cells were cultured in a medium formulated from low-glucose DMEM (Thermo Fisher, MA, USA) and 10% fetal bovine serum (FBS, Gibco, MA, USA) and then enriched for plastic adherence in a humidified 2% O_2_ and 5% CO_2_ incubator. A standard miltirone sample for analysis (HPLC≥98%) (Yuanye Biology, Shanghai, China) was extracted from *Salvia miltiorrhizae* Radix. Miltirone was dissolved in DMSO (Beyotime, Shanghai, China) at final concentrations varying from 10 to 60 μM. VX-765 was purchased from Sigma-Aldrich (Darmstadt, Germany), and the working concentration of VX-765 was 20 μM. The KIRC cells were pretreated with VX-765 for 4 h, subsequently treated with miltirone for 24 h/48 h, and subjected to cell assays.

### Cell viability

Cell proliferation or cytotoxicity assays were evaluated using MTT Cell Growth Assay Kit (Sigma-Aldrich). The cells treated with VX-765 and/or miltirone were seeded in 96-well plates (6,000 cells/well) and incubated for 24 h/48 h. Then, 10 μL of MTT was added to each well, and the wells were incubated for 4 h to dissociate MTT. Each well was added with 50 μL of isopropyl alcohol and incubated for 1 h. Next, we measured the absorbance at 570 nm using a microplate reader.

### Flow cytometry

Cell pyroptosis was evaluated using eBioscience™ Annexin V-FITC Apoptosis Detection Kit (Invitrogen, MA, USA). The cells treated with VX-765 and/or miltirone were washed with cold PBS and gently shaken. A total of 200 µL binding buffer (1×) was utilized to resuspend the cells with a cell density of 2–5 × 10^5^/mL. Then, 5 µL Annexin V-FITC, along with 195 µL of cell suspension, was incubated for 10 min at room temperature and washed with 200 µL binding buffer (1×). Next, the cells were incubated with 10 µL propidium iodide (20 µg/mL) for 1 h in the dark. FACS analysis was performed to assess cell pyroptosis percentages.

### ELISA assay

The release of IL-1β and IL-18 cytokines was detected with ElaBoX™ Human IL-1β ELISA Kit (Solarbio, Beijing, China) and ElaBoX™ Human IL-1β ELISA Kit (Solarbio). The culture media following treatment with VX-765 and/or miltirone were collected and subjected to centrifugation to remove cellular debris. The levels of IL-1β and IL-18 present in the culture medium were quantified using ELISA kits and analyzed with a microplate reader.

### Western blot

Total protein was extracted from treated A498 cells using RIPA buffer supplemented with a protease and phosphatase inhibitor cocktail. The protein concentration was determined using a bicinchoninic acid (BCA) assay kit. Equal amounts of protein were separated by SDS-PAGE and subsequently transferred onto PVDF membranes. The membranes were blocked with 5% non-fat milk in Tris-buffered saline containing 0.1% Tween 20 (TBST) for 1 h at room temperature and then incubated with specific primary antibodies diluted in blocking buffer overnight at 4 °C. The primary antibodies used in this study included anti-NLRP3 (1:1,000, Abcam, ab263899), anti-AIM2 (1:1,000, Cell Signaling Technology, #12948), anti-pro-caspase-3 (1:1,000, Cell Signaling Technology, #9662), anti-cleaved-caspase-3 (1:1,000, Cell Signaling Technology, #9664), anti-GSDMD-FL (1:1,000, Abcam, ab219800), anti-cleaved N-terminal GSDMD (1:1,000, Abcam, ab215203), and anti-β-actin (1:5,000, Cell Signaling Technology, #4967). A horseradish peroxidase (HRP)-conjugated goat anti-rabbit or anti-mouse immunoglobulin G (IgG (H + L)) was used as the secondary antibody. After thorough washing with TBST, protein bands were visualized using an enhanced chemiluminescence (ECL) substrate and imaged. β-Actin was used as a loading control for normalization.

### Statistics analysis

All of the cell experimental assays were performed in triplicate. The data were collected and statistically evaluated using GraphPad Prism software. The results are presented as mean ± standard error of the mean (SEM). To determine significant differences between groups, either a *t*-test or a one-way analysis of variance (ANOVA) was performed, followed by Tukey’s multiple comparison test. A *p*-value <0.05 was deemed statistically significant.

## Results

### mRNA and protein expression of NLRP3 and AIM2 in pan-cancer

**Figure 1:**
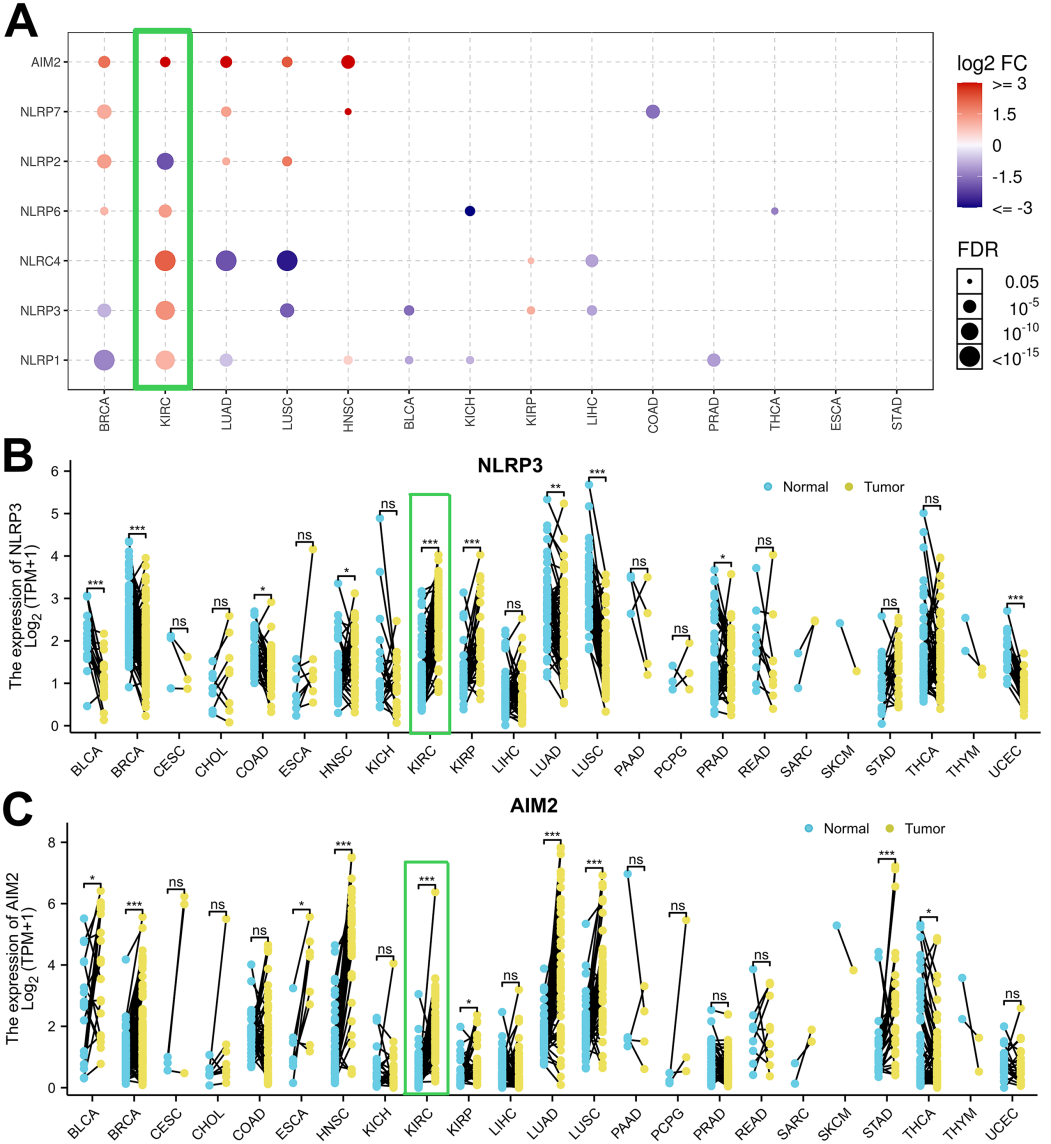
Assessment of mRNA/protein expression levels for NLRP3 and AIM2 among pan-cancer. **(A)** The GSCALite platform was employed for the analysis of NLR family and AIM2 mRNA levels, comparing tumor samples with normal tissues from GTEx. **(B, C)** Dot plots graphically represented the expression in mRNA expression levels of NLRP3 and AIM2.

**Figure 2:**
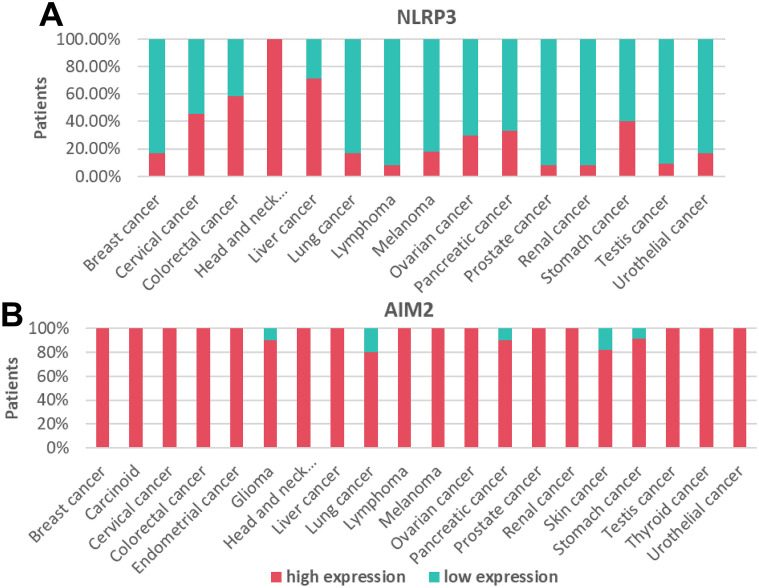
Assessment of protein expression for NLRP3 and AIM2 among pan-cancer. **(A, B)** The analysis depicts the proportion of patients exhibiting either an elevated or a diminished expression of NLRP3 and AIM2 protein expression in pan-cancer. Columns shaded in red indicate increased protein expression levels, while those in green denote reduced levels of protein expression.

To profile the mRNA expression landscape of pan-cancer, we analyzed the transcriptional expression atlas in 14 common cancers. GSCALite database collects clinical data from NCI Genomic Data Commons and provides mRNA differential expression with paired tumor and normal samples. **Figure 1A** shows that AIM2 mRNAs were upregulated among BRCA, KIRC, LUAD, LUSC, and HNSC. However, the expression of NLRP3 gene varies greatly in different tumors. To understand the differential expression, we performed an analysis in TCGA tumor tissues and adjacent normal tissues and looked for changes in NLR mRNA levels. NLR genes were upregulated in KIRC and KIRP and were downregulated in BLCA, BRCA HNSC, LUAD, LUSC, and UCEC compared with para-carcinoma tissues (**Figure 1B**). Moreover, **Figure 1C** shows that BLCA, BRCA, HNSC, and KIRC have a higher expression of AIM2 levels compared with human normal tissues. Furthermore, **Figure 2A** indicates that NLRP3 was moderately stained by specific antibodies in most cancer patients. Importantly, AIM2 protein was widely highly expressed in pan-cancer (**Figure 2B**).

### Genetic alterations of NLRP3 and AIM2 among pan-cancer

**Figure 3:**
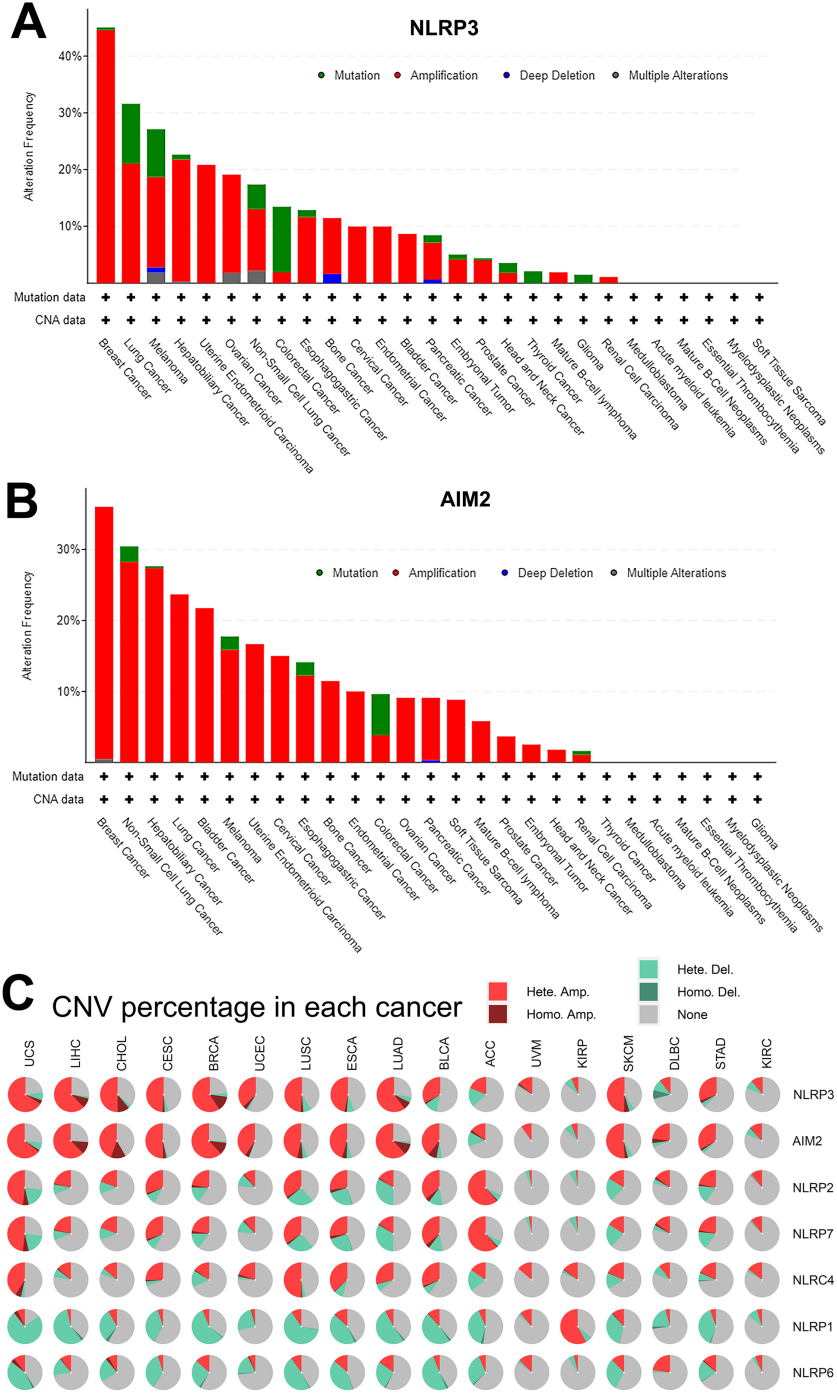
Genetic alterations of NLRP3 and AIM2 among pan-cancer. **(A, B)** cBioPortal analysis for the alteration frequency of NLRP3 and AIM2 in pan-cancer. **(C)** CNV percentage of NLRs and AIM2 in pan-cancer.

We next assessed whether the differential expression of NLR family and AIM2 was preserved in the genetic structure, focusing on genetic alteration frequency, copy number variations (CNV), and methylation. As displayed in **Figures 3A, B**, the cBioPortal analysis demonstrated that the alteration frequency of NLRP3 and AIM2 was obviously high in pan-cancer. Moreover, the genomic amplification alteration of NLRP3 and AIM2 was shown in renal cell carcinoma (**Figures 3A, B**). To assess the correlation between CNV percentage and NLR family in pan-cancer, we performed Gene Set Cancer Analysis (GSCA) to further explore genomic mutations. As indicated in **Figure 3C**, UCS, LIHC, CHOL, CESA, and BRCA had high CNV percentage in each cancer dataset. The mutation patterns of NLRP3 and AIM2 in pan-cancer were highly similar (**Figure 3C**).

### Correlation between NLRP3/AIM2 expression levels and immune microenvironment among pan-cancer

**Figure 4:**
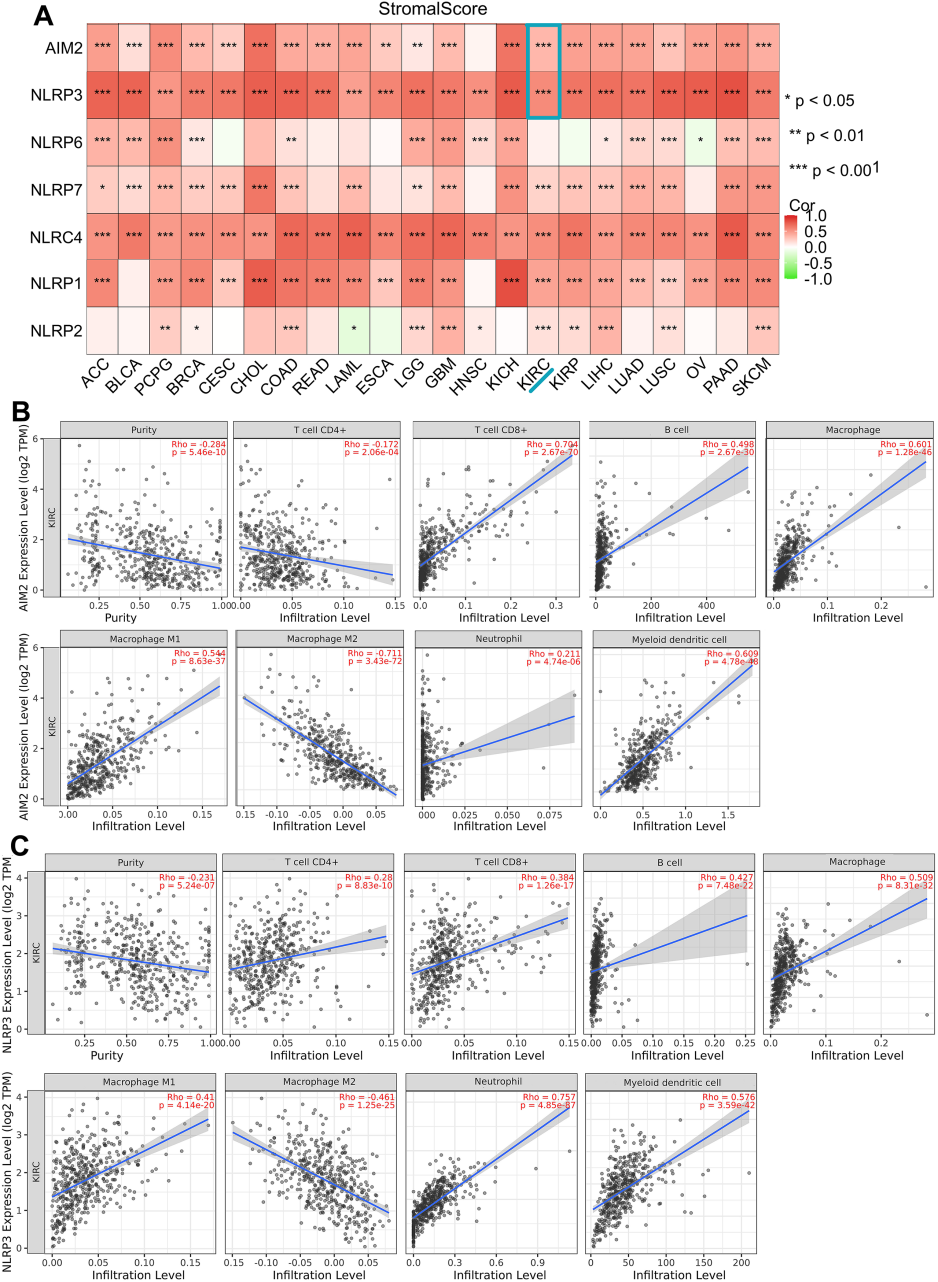
Correlation between NLR family expression and immune microenvironment in pan-cancer. **(A)** StromalScore algorithm was utilized to investigate the association between NLRs/AIM2 and immune response in pan-cancer. **(B, C)** Immune infiltration analysis of AIM2 and NLRP3 in KIRC. * p < 0.05, ** p < 0.01, *** p < 0.001.

There are two major types of non-tumor components in the tumor microenvironment—immune cells and stromal cells. Existing research recognized the critical roles played by immune cells and stromal cells in tumor diagnosis and prognostic evaluation. In StromalScore analysis, NLRP3 and AIM2 have positive correlations with stromal components among pan-cancers (*p* < 0.001, **Figure 4A**). This indicated that NLRP3 and AIM2 significantly regulated the tumor microenvironment among all the cancer types. To investigate the involvement of NLRP3 and AIM2 in the KIRC tumor microenvironment, we utilized TIMER algorithms to assess the potential Spearman coefficient between immune infiltration level and pyroptosis-related gene expression. A statistically positive correlation was observed between AIM2 expression and the immune infiltration of CD8+ T cells, B cells, macrophage, and myeloid dendritic cells (Rho > 0.5, *p* < 0.001, **Figure 4B**). NLRP3 expression showed a high correlation with CD4+ T cells, CD8+ T cells, B cells, M1 macrophages, neutrophils, and myeloid dendritic cells. However, the NLRP3 level was negatively correlated with macrophage M2 cells (*p* < 0.001, **Figure 4C**). Moreover, the result indicated a close correlation between NLRP3/AIM2 and the immune cells in the KIRC samples.

### Co-expression and functional analysis of pyroptosis-related genes in KIRC

**Figure 5:**
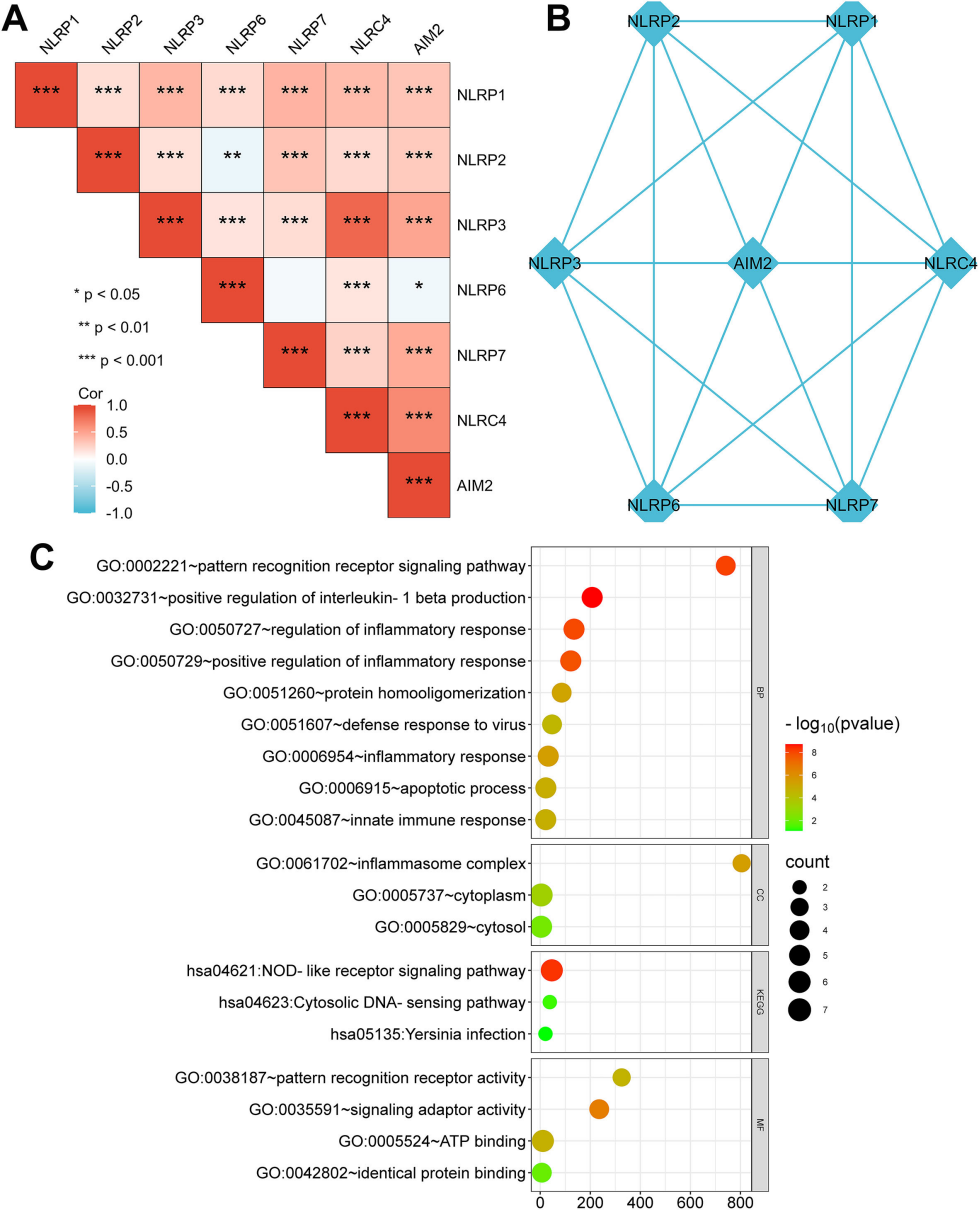
Co-expression and functional analysis of pyroptosis-related genes in KIRC. **(A)** Correlation between the NLR family and AIM2 gene expression in KIRC tissues. *p < 0.05, **p < 0.01, ***p < 0.001. **(B)** The interaction of NLRs/AIM2 protein was illustrated with protein–protein interaction (PPI) network. The network node represents proteins. The edge represents the predicted functional associations. **(C)** Enrichment analysis using GO gene sets and KEGG for NLRs/AIM2 genes.

To identify how NLRP3 and AIM2 regulate the immune response, we carried out an analysis of the expression correlation among a set of seven pyroptosis-related genes in KIRC. The mRNA expression level of AIM2 was obviously correlated with NLRP3 expression in KIRC (*R* = 0.81, *p* < 0.001, **Figure 5A**). The PPI network of the seven pyroptosis-related genes is indicated in **Figure 5B**. Then, we performed GO enrichment and KEGG analysis with the NLR family and AIM2 to explore the underlying regulatory function. The results suggested that the NLR family and AIM2 activated interleukin-1 beta production and cellular inflammatory response (**Figure 5C**). Moreover, the KEGG pathway data indicated that the NLR family and AIM2 were involved in the NOD-like receptor signaling pathway (**Figure 5C**).

### Correlation analysis between immune subtype and NLRP3/AIM2 expression levels

**Figure 6:**
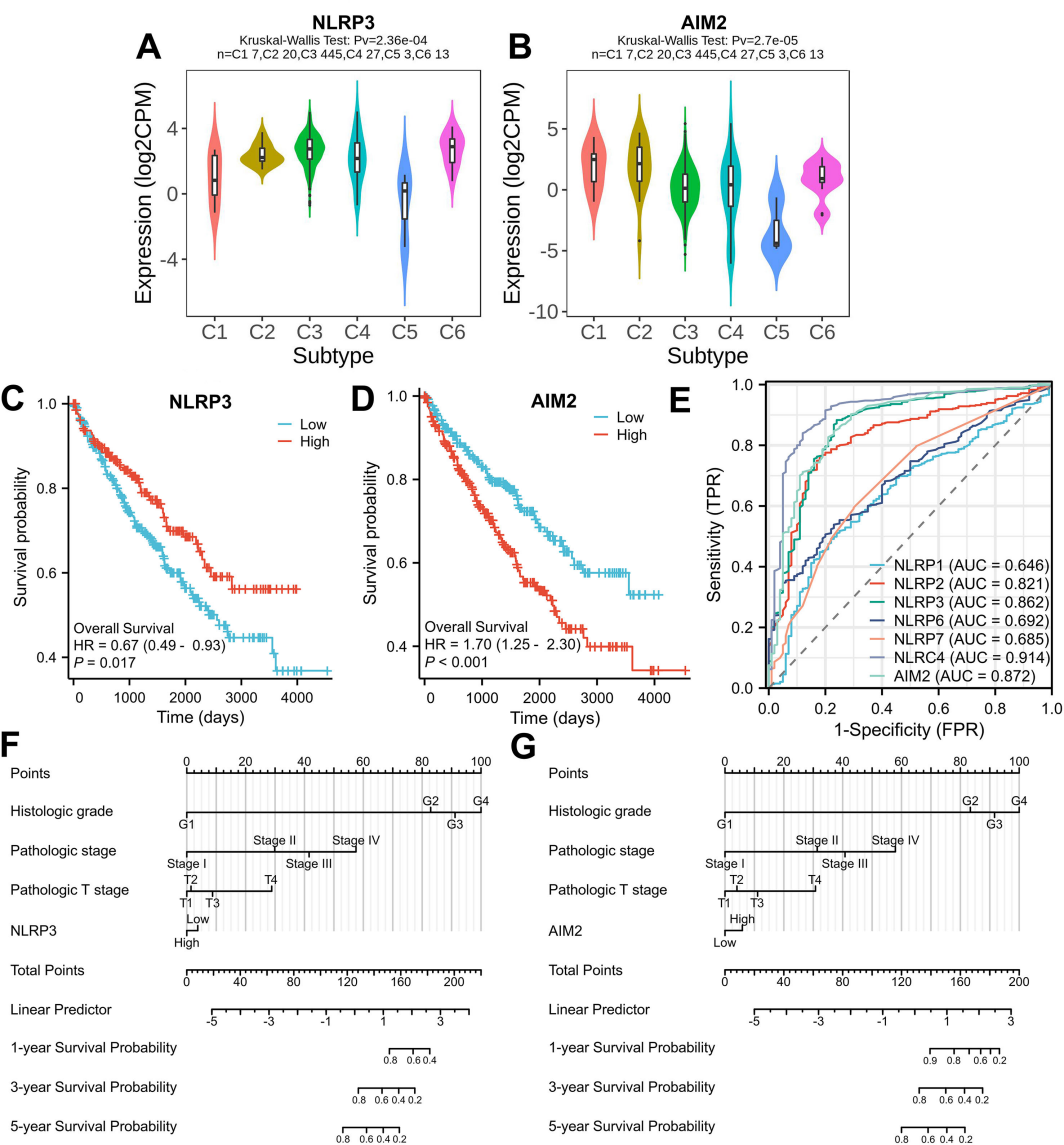
Prognostic value of NLRs and AIM2 in KIRC. **(A, B)** Correlation analysis between immune subtype and NLRP3/AIM2 gene expression. **(C, D)** Kaplan–Meier curves of overall survival data in KIRC patients based on the levels of NLRP3 and AIM2. **(E)** ROC curve analysis of NLRs/AIM2 showing the predictive efficiency. **(F, G)** Nomogram combining risk score for predicting 1-, 3-, and 5-year overall survival probability in KIRC patients.

To further assess the immune function of NLRP3/AIM2 in KIRC, correlation coefficients between immune subtype and NLRP3/AIM2 expression were analyzed by tumor–immune interaction analysis. As displayed in **Figure 6A**, we observed an increased expression of NLRP3 in C3 (inflammatory) and C6 (TGF-beta dominant) immune subtypes. **Figure 6B** indicated that the expression of AIM2 was obviously related to immune subtype, including C1 (wound healing) and C2 (IFN-gamma dominant) in KIRC.

### Prognostic value of NLRP3 and AIM2 in KIRC

Next, Kaplan–Meier survival analysis was performed to elucidate the prognostic roles of NLR and AIM2 expression in KIRC patients. According to the mRNA expression of NLR genes, the KIRC clinical data were divided into two groups, namely high expression (50%) and low expression (50%), for KM analysis. As shown in **Figures 6C, D**, the high expression of AIM2 (*p* < 0.001) correlated with worse prognosis in patients with KIRC. Moreover, the ROC curves determined the risk score of NLRP3 and AIM2 prognosis. NLRP3 and AIM2 had a high accuracy in predicting patient outcomes, and the corresponding AUC values of NLRP3 and AIM2 were 0.862 and 0.872, respectively (**Figure 6E**). Additionally, survival analysis indicated that AIM2 expression was significant in univariate Cox regression. **Figures 6F, G** show that the clinical factors and NLRP3/AIM2 expression levels could be combined to establish a nomogram for the prediction of the 1-, 3-, and 5-year overall survival.

### Miltirone inhibited KIRC cell proliferation *in vitro*

**Figure 7:**
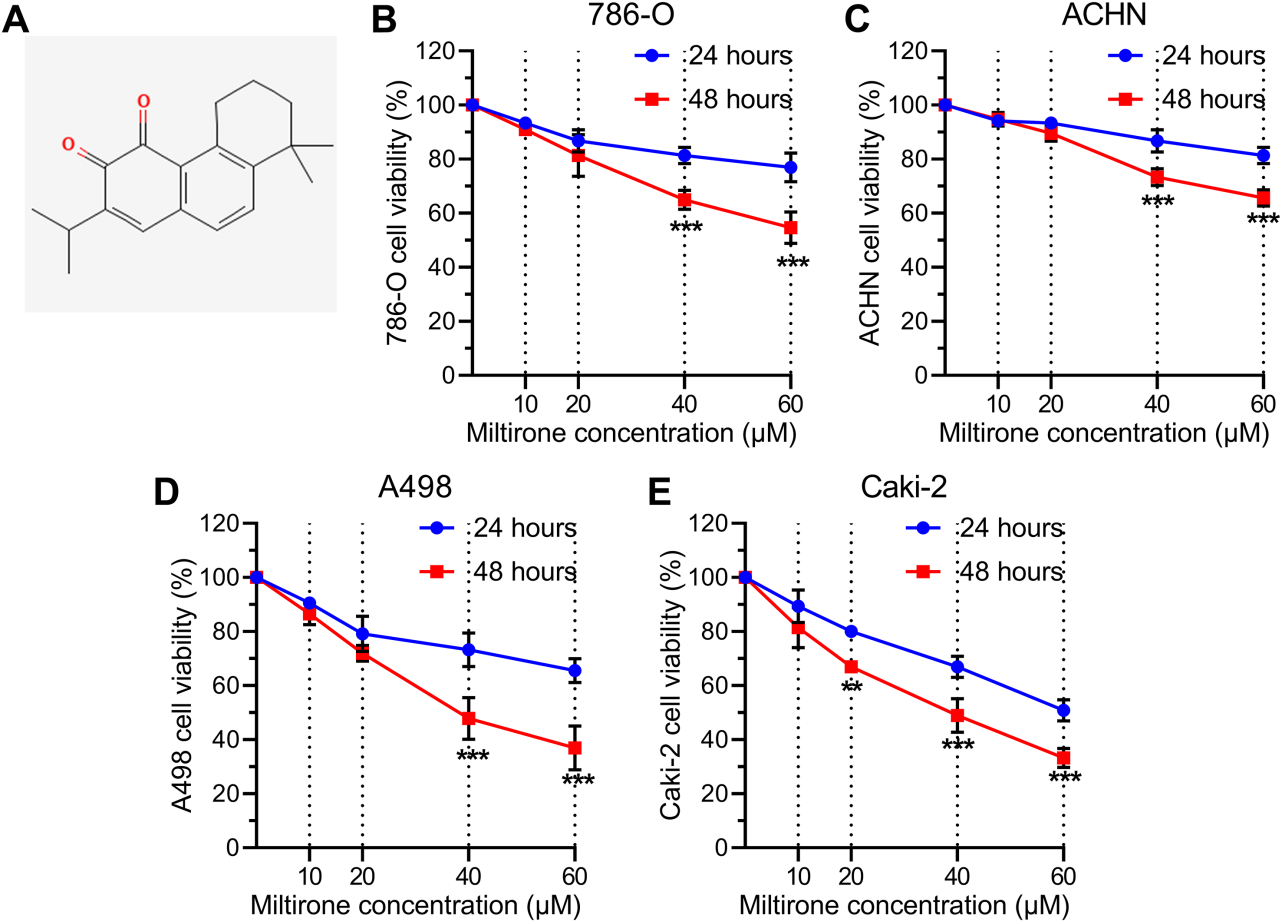
Miltirone significantly inhibited KIRC cell proliferation. **(A)** Chemical structure of miltirone. **(B–E)** Cell viability of KIRC cells (786-O, ACHN, A498, and Caki-2) treated with different concentrations (10, 20, 40, and 60 μM) of miltirone. **p < 0.01, ***p < 0.001.

**Figure 7A** illustrates the chemical structure of miltirone. To assess whether miltirone has the potential to inhibit the proliferation of KIRC cells, we developed an *in vitro* KIRC model using four cell lines: 786-O, ACHN, A498, and Caki-2. By performing the MTT assay, we observed that miltirone significantly suppressed the proliferation of KIRC cells. The inhibitory effects of miltirone were both dose-dependent and time-dependent, as evidenced by the reduced proliferation of four KIRC cell lines compared to the blank groups (0, 40, or 60 μM, 24 h/48 h incubation) (**Figures 7B–E**). Our findings indicated that A498 and Caki-2 cells were sensitive to miltirone treatment, leading us to select these two cell lines for further experiments.

### Miltirone activated KIRC cell pyroptosis by regulating the NLRP3/AIM2/caspase-3/GSDMD axis

**Figure 8:**
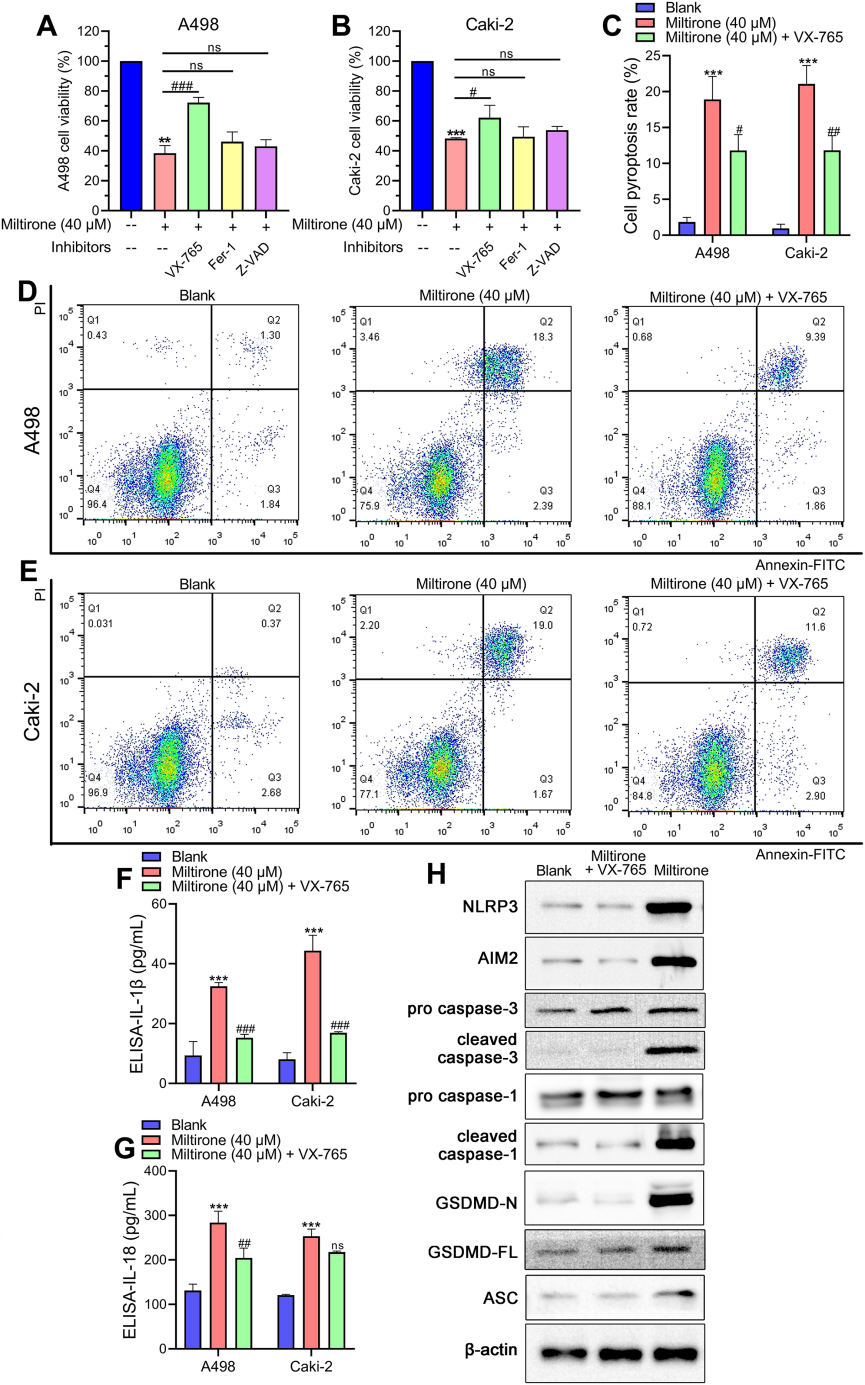
Miltirone activated KIRC cell pyroptosis by regulating the NLRP3/AIM2/caspase-3/GSDMD axis. A498 and Caki-2 cells were pretreated with VX-765 (pyroptosis inhibitor), Fer-1 (ferroptosis inhibitor), or Z-VAD (apoptosis inhibitor) for 4 h and then incubated with 40 μM miltirone for 48 (h) **(A, B)** Cell viabilities in A498 and Caki-2 cells were assessed utilizing MTT assay. **(C–E)** Pyroptosis was detected using flow cytometry. **(F, G)** ELISA assays were performed to detect the release of IL-1β and IL-18 cytokines. ***p < 0.001 compared with the blank group. ^#^*p* < 0.05, ^##^*p* < 0.01, ^###^*p* < 0.001 compared with the miltirone (40 μM) group. **(H)** Western blot assessment of the protein expression of NLRP3, AIM2, caspase-1, caspase-3, and GSDMD in A498 cells.

To explore how miltirone functions in the inhibition of KIRC cell proliferation, we assessed the impact of different cell death inhibitors on KIRC cells. Miltirone showed an obvious suppression of A498 and Caki-2 cell growth. Importantly, the proliferation inhibition was reversed by a pyroptosis inhibitor (VX-765) and not influenced by ferroptosis or apoptosis inhibitors (Fer-1, Z-VAD) (**Figures 8A, B**). To elucidate whether miltirone induces pyroptosis in KIRC cells, we performed flow cytometry analysis by utilizing Annexin V/FITC staining. The flow cytometry results showed that miltirone treatment increased the Annexin V-stained and FITC-labeled cells, indicating the damage of the cell membrane (*p* < 0.001, **Figures 8C–E**). The pyroptosis increase was reversed by VX-765 in miltirone-treated A498 and Caki-2 cells (*p* < 0.05, *p* < 0.01, **Figures 8C–E**). The ELISA assay demonstrated a notable release of IL-1β and IL-18 cytokines in the culture medium of A498 and Caki-2 cells treated with miltirone. This release was significantly inhibited by the pyroptosis inhibitor VX-765 (*p* < 0.01, *p* < 0.001, **Figures 8F, G**). The Western blot results indicated that after the miltirone treatment, the NLRP3 and AIM2 protein expression levels were significantly increased compared with the blank group in A498 cells (**Figure 8H**). The treatment of miltirone induced the cleavage of caspase 3, caspase 1, GSDMD, and apoptosis-associated speck-like protein containing a CARD (ASC) speck expression in KIRC cells (**Figure 8H**). We hypothesized that GSDMD might be essential for the miltirone-induced pyroptosis in KIRC. Furthermore, the pyroptosis inhibitor (VX-765) reversed the increased expression levels of pyroptosis-related proteins induced by miltirone (**Figure 8H**). These results suggest that VX-765 attenuates miltirone-induced pyroptosis and proliferation inhibition in KIRC cells.

## Discussion

Our study focuses on the comprehensive bioinformatic analysis of pyroptosis-related genes, including mRNA/protein expression, genetic alterations, methylation patterns, and immune infiltration levels among pan-cancers. The results have unveiled significant insights into the role of NLRP3 and AIM2 in tumorigenesis and immune regulation and suggested the potential values of pyroptosis-related genes as diagnostics and therapeutic targets.

AIM2 inflammasome (17) and NLR proteins (18) are important components in innate immune response, playing pivotal roles in tumors. The AIM2 inflammasome is involved in the progression and immune evasion of several cancers, including melanoma, colorectal cancer, and glioma (13, 19–22). Several studies about AIM2 have elucidated that AIM2 inflammasome activation has a pivotal role in inducing pyroptosis, thereby inhibiting tumor growth (23–25). The result shown in our study demonstrated that the upregulation of AIM2 in BRCA, KIRC, LUAD, LUSC, and HNSC, as well as the high expression levels of NLR family genes, suggested a nuanced role of pyroptosis-related genes in cancer progression and immune evasion. Our findings align with previous reports indicating the involvement of AIM2 in DNA damage response and inflammation, acting as a double-edged sword in cancer biology (26). AIM2 can promote genomic stability and prevent tumorigenesis (27), and its activation leads to chronic inflammation (28).

The NLR family, which is a pattern recognition receptor, has been shown to modulate immune response within the tumor microenvironment through their involvement in cytokine production, cell death pathways, and autophagy (29–31). Research has demonstrated that NLRs can trigger pyroptosis by activating caspase-1, subsequently leading to the cleavage of gasdermin D and the release of inflammatory cytokines such as IL-1β and IL-18 (13, 32). Our study revealed that the expression patterns, genetic variations, and epigenetic modifications of NLRs significantly influenced immune cell infiltration and patient prognosis. The results provided converging evidence for the potential diagnostic or prognostic values of NLRP3 in pan-cancer.

A detailed tumor microenvironment analysis is critical to identify the function of pyroptosis-related genes among pan-cancers. Here we firstly described a significant correlation between NLRP3/NLRC4/AIM2 expression and cell infiltration at the immune level in KIRC. This finding suggested that pyroptosis-related genes modulate the tumor microenvironment of KIRC, potentially affecting tumor immunity and response to therapy. The positive correlation between AIM2 expression and immune infiltration, especially CD8+ T cells, indicated the potential of AIM2 as a marker of immune activity and a target for immunotherapy.

Miltirone exhibits notable anti-inflammatory properties (33) and has been shown to induce tumor cell death (7), which has led to a growing interest in its potential anticancer effects—for instance, miltirone was reported to be an effective agent for pro-apoptosis in colon cancer with p53-dependent and ROS-dependent pathways (5). Zhu’s study indicated that miltirone promoted lung cancer cell apoptosis and reversed cisplatin resistance by increasing the protein expression of Bax and p53 (6). In addition, it has been reported that miltirone inhibits the proliferation of human colon cancer cells and activates caspase-3 to induce cell pyroptosis (7). An *in vitro* experiment revealed that miltirone exhibited the ability to suppress the proliferation of KIRC cells, aligning with previous research that highlights miltirone’s anticancer properties. Moreover, we examined the expression of pyroptosis-related biomarkers (NLRP3, AIM2, and GSDMD) to further study the mechanism of miltirone-induced pyroptosis by suppressing GSDMD expression in KIRC. Surprisingly, the pyroptosis-specific inhibitor VX-765 demonstrates a protective effect in KIRC cells.

The relationship between NLRP3 and AIM2 in KIRC cells appears to be characterized by parallel activation rather than a strictly hierarchical cascade. Given that miltirone treatment led to the concurrent upregulation of both sensors, it is plausible that miltirone serves as a pleiotropic stimulus, potentially triggering mitochondrial ROS (activating NLRP3) and cytosolic DNA release (activating AIM2) simultaneously. These parallel signals converge on the cleavage of GSDMD, thereby executing the pyroptotic program. Despite the robust evidence that miltirone induces pyroptosis via the NLRP3/AIM2/GSDMD axis, the specific mechanism requires further clarification. Specifically, the direct molecular target, the primary protein to which miltirone binds, remains unidentified. While our functional assays clearly show that miltirone triggers a specific signaling cascade involving the NLRP3/AIM2/GSDMD axis, identifying its direct target through structural biology remains a priority for our future research.

In general, this study provided a comprehensive overview of the role of NLRP3 and AIM2 in KIRC, highlighting their significance in cancer biology, immune regulation, and clinical prognosis. The prognostic value of NLRP3 and AIM2 expression in KIRC, as demonstrated by the Kaplan–Meier survival analysis and ROC curves, emphasized the potential of pyroptosis-related genes as biomarkers for patient outcome prediction. We revealed that miltirone induces pyroptosis through the NLRP3/AIM2/GSDMD pathway in KIRC. Our findings provide novel insights into future understanding of the mechanism of miltirone in KIRC treatment. Further research is required to focus on xenograft tumor models and clinical studies.

## Data Availability

The datasets presented in this study can be found in online repositories. The names of the repository/repositories and accession number(s) can be found in the article/supplementary material.

## References

[1] Hsieh JJ Purdue MP Signoretti S Swanton C Albiges L Schmidinger M . Renal cell carcinoma. Nat Rev Dis Primers. (2017) 3:17009. doi: 10.1038/nrdp.2017.9, PMID: 28276433 PMC5936048

[2] Xu J Hu Z Cao H Zhang H Luo P Zhang J . Multi-omics pan-cancer study of cuproptosis core gene FDX1 and its role in kidney renal clear cell carcinoma. Front Immunol. (2022) 13:981764. doi: 10.3389/fimmu.2022.981764, PMID: 36605188 PMC9810262

[3] Chevrier S Levine J Zanotelli V Silina K Schulz D Bacac M . An immune atlas of clear cell renal cell carcinoma. Cell. (2017) 169:736–749.e18. doi: 10.1016/j.cell.2017.04.016, PMID: 28475899 PMC5422211

[4] Zheng G Fang Z Lin Z Guan G . Miltirone induces GSDME-dependent pyroptosis in colorectal cancer by activating caspase 3. Heliyon. (2024) 10:e36603. doi: 10.1016/j.heliyon.2024.e36603, PMID: 39262975 PMC11388397

[5] Wang L Hu T Shen J Zhang L Li L-f RL-Y Chan . Miltirone induced mitochondrial dysfunction and ROS-dependent apoptosis in colon cancer cells. Life Sci. (2016) 151:224–34. doi: 10.1016/j.lfs.2016.02.083, PMID: 26969764

[6] Zhu Z . Miltirone-induced apoptosis in cisplatin-resistant lung cancer cells through upregulation of p53 signaling pathways. Oncol Lett. (2018) 15:8841–6. doi: 10.3892/ol.2018.8440, PMID: 29928326 PMC6004674

[7] Zhang X Zhang P An L Sun N Peng L Tang W . Miltirone induces cell death in hepatocellular carcinoma cell through GSDME-dependent pyroptosis. Acta Pharm Sin B. (2020) 10:1397–413. doi: 10.1016/j.apsb.2020.06.015, PMID: 32963939 PMC7488361

[8] Yang F Bettadapura S Smeltzer M Zhu H Wang S . Pyroptosis and pyroptosis-inducing cancer drugs. Acta Pharmacol Sin. (2022) 43:2462–73. doi: 10.1038/s41401-022-00887-6, PMID: 35288674 PMC9525650

[9] Rao Z Zhu Y Yang P Chen Z Xia Y Qiao C . Pyroptosis in inflammatory diseases and cancer. Theranostics. (2022) 12:4310–29. doi: 10.7150/thno.71086, PMID: 35673561 PMC9169370

[10] Vasudevan SO Behl B Rathinam VA . Pyroptosis-induced inflammation and tissue damage. Semin Immunol. (2023) 69:101781. doi: 10.1016/j.smim.2023.101781, PMID: 37352727 PMC10598759

[11] Wei X Xie F Zhou X Wu Y Yan H Liu T . Role of pyroptosis in inflammation and cancer. Cell Mol Immunol. (2022) 19:971–92. doi: 10.1038/s41423-022-00905-x, PMID: 35970871 PMC9376585

[12] Xia X Wang X Cheng Z Qin W Lei L Jiang J . The role of pyroptosis in cancer: pro-cancer or pro-”host”? Cell Death Dis. (2019) 10:650. doi: 10.1038/s41419-019-1883-8, PMID: 31501419 PMC6733901

[13] Man SM Kanneganti TD . Regulation of inflammasome activation. Immunol Rev. (2015) 265:6–21. doi: 10.1111/imr.12296, PMID: 25879280 PMC4400844

[14] Platnich JM Muruve DA . NOD-like receptors and inflammasomes: A review of their canonical and non-canonical signaling pathways. Arch Biochem Biophys. (2019) 670:4–14. doi: 10.1016/j.abb.2019.02.008, PMID: 30772258

[15] Tian X Zhang S Zhang Q Kang L Ma C Feng L . Resveratrol inhibits tumor progression by down-regulation of NLRP3 in renal cell carcinoma. J Nutr Biochem. (2020) 85:108489. doi: 10.1016/j.jnutbio.2020.108489, PMID: 32827663

[16] Wang K Xu T Ruan H Xiao H Liu J Song Z . LXRα promotes cell metastasis by regulating the NLRP3 inflammasome in renal cell carcinoma. Cell Death Dis. (2019) 10:159. doi: 10.1038/s41419-019-1345-3, PMID: 30770793 PMC6377709

[17] Choubey D . Absent in melanoma 2 proteins in the development of cancer. Cell Mol Life Sci. (2016) 73:4383–95. doi: 10.1007/s00018-016-2296-9, PMID: 27328971 PMC11108365

[18] Sharma BR Kanneganti TD . NLRP3 inflammasome in cancer and metabolic diseases. Nat Immunol. (2021) 22:550–9. doi: 10.1038/s41590-021-00886-5, PMID: 33707781 PMC8132572

[19] Sharma BR Karki R Kanneganti TD . Role of AIM2 inflammasome in inflammatory diseases, cancer and infection. Eur J Immunol. (2019) 49:1998–2011. doi: 10.1002/eji.201848070, PMID: 31372985 PMC7015662

[20] Man SM Karki R Kanneganti TD . AIM2 inflammasome in infection, cancer, and autoimmunity: Role in DNA sensing, inflammation, and innate immunity. Eur J Immunol. (2016) 46:269–80. doi: 10.1002/eji.201545839, PMID: 26626159 PMC4758349

[21] Chen J Wang Z Yu S . AIM2 regulates viability and apoptosis in human colorectal cancer cells via the PI3K/Akt pathway. Onco Targets Ther. (2017) 10:811–7. doi: 10.2147/OTT.S125039, PMID: 28243117 PMC5315344

[22] Chen D Le S Hutchinson T Calinescu A Sebastian M Jin D . Tumor Treating Fields dually activate STING and AIM2 inflammasomes to induce adjuvant immunity in glioblastoma. J Clin Invest. (2022) 132:e149258. doi: 10.1172/JCI149258, PMID: 35199647 PMC9012294

[23] Gao J Peng S Shan X Deng G Shen L Sun J . Inhibition of AIM2 inflammasome-mediated pyroptosis by Andrographolide contributes to amelioration of radiation-induced lung inflammation and fibrosis. Cell Death Dis. (2019) 10:957. doi: 10.1038/s41419-019-2195-8, PMID: 31862870 PMC6925222

[24] Meng J Li N Liu X Qiao S Zhou Q Tan J . NLRP3 attenuates intraocular inflammation by inhibiting AIM2-mediated pyroptosis through the phosphorylated salt-inducible kinase 1/sterol regulatory element binding transcription factor 1 pathway. Arthritis Rheumatol. (2023) 75:842–55. doi: 10.1002/art.42420, PMID: 36529965

[25] Zhang X Liu R . Pyroptosis-related genes GSDMB, GSDMC, and AIM2 polymorphisms are associated with risk of non-small cell lung cancer in a Chinese Han population. Front Genet. (2023) 14:1212465. doi: 10.3389/fgene.2023.1212465, PMID: 37359371 PMC10287965

[26] Lozano-Ruiz B González-Navajas JM . The emerging relevance of AIM2 in liver disease. Int J Mol Sci. (2020) 21:6535. doi: 10.3390/ijms21186535, PMID: 32906750 PMC7555176

[27] Wilson JE Petrucelli AS Chen L Koblansky AA Truax AF Oyama Y . Inflammasome-independent role of AIM2 in suppressing colon tumorigenesis via DNA-PK and Akt. Nat Med. (2015) 21:906–13. doi: 10.1038/nm.3908, PMID: 26107252 PMC4529369

[28] Onódi Z Ruppert M Kucsera D Sayour A Tóth V Koncsos G . AIM2-driven inflammasome activation in heart failure. Cardiovasc Res. (2021) 117:2639–51. doi: 10.1093/cvr/cvab202, PMID: 34117866

[29] Zhang Y Song G Lal N Nagalakshmi U Li Y Zheng W . TurboID-based proximity labeling reveals that UBR7 is a regulator of N NLR immune receptor-mediated immunity. Nat Commun. (2019) 10:3252. doi: 10.1038/s41467-019-11202-z, PMID: 31324801 PMC6642208

[30] Biasizzo M Kopitar-Jerala N . Interplay between NLRP3 inflammasome and autophagy. Front Immunol. (2020) 11:591803. doi: 10.3389/fimmu.2020.591803, PMID: 33163006 PMC7583715

[31] Xu J Núñez G . The NLRP3 inflammasome: activation and regulation. Trends Biochem Sci. (2023) 48:331–44. doi: 10.1016/j.tibs.2022.10.002, PMID: 36336552 PMC10023278

[32] Tuladhar S Kanneganti TD . NLRP12 in innate immunity and inflammation. Mol Aspects Med. (2020) 76:100887. doi: 10.1016/j.mam.2020.100887, PMID: 32838963 PMC9375713

[33] Wang H Gu J Hou X Chen J Yang N Liu Y . Anti-inflammatory effect of miltirone on inflammatory bowel disease via TLR4/NF-κB/IQGAP2 signaling pathway. BioMed Pharmacother. (2017) 85:531–40. doi: 10.1016/j.biopha.2016.11.061, PMID: 27903427

